# Review on the Biogenesis of Platelets in Lungs and Its Alterations in SARS-CoV-2 Infection Patients

**DOI:** 10.1155/2023/7550197

**Published:** 2023-02-27

**Authors:** Balasundaram Nandhini, Yacobu Sureshraj, Mohandass Kaviya, Thangavelu Sangeetha, Kathirvel Bharathi, Balasubramanian Balamuralikrishnan, Pappuswamy Manikantan, Meyyazhagan Arun, Kuchi Bhotla Haripriya, Pushparaj Karthika, Subramaniam Kalidass, Arumugam Vijaya Anand

**Affiliations:** ^1^Department of Human Genetics and Molecular Biology, Bharathiar University, Coimbatore, Tamil Nadu, India; ^2^Department of Food Science and Biotechnology, Sejong University, Seoul, Republic of Korea; ^3^Department of Life Sciences, Christ Deemed to be University, Bengaluru, India; ^4^Department of Zoology, Avinashilingam Institute for Home Science and Higher Education for Women, Coimbatore, Tamil Nadu, India; ^5^Department of Animal Science, Manonmaniam Sundaranar University, Tirunelveli, Tamil Nadu, India

## Abstract

Thrombocytes (platelets) are the type of blood cells that are involved in hemostasis, thrombosis, etc. For the conversion of megakaryocytes into thrombocytes, the thrombopoietin (TPO) protein is essential which is encoded by the *TPO* gene. *TPO* gene is present in the long arm of chromosome number 3 (3q26). This TPO protein interacts with the c-Mpl receptor, which is present on the outer surface of megakaryocytes. As a result, megakaryocyte breaks into the production of functional thrombocytes. Some of the evidence shows that the megakaryocytes, the precursor of thrombocytes, are seen in the lung's interstitium. This review focuses on the involvement of the lungs in the production of thrombocytes and their mechanism. A lot of findings show that viral diseases, which affect the lungs, cause thrombocytopenia in human beings. One of the notable viral diseases is COVID-19 or severe acute respiratory syndrome caused by *SARS*-associated *coronavirus 2* (SARS-CoV-2). SARS-CoV-2 caused a worldwide alarm in 2019 and a lot of people suffered because of this disease. It mainly targets the lung cells for its replication. To enter the cells, these virus targets the angiotensin-converting enzyme-2 (ACE-2) receptors that are abundantly seen on the surface of the lung cells. Recent reports of COVID-19-affected patients reveal the important fact that these peoples develop thrombocytopenia as a post-COVID condition. This review elaborates on the biogenesis of platelets in the lungs and the alterations of thrombocytes during the COVID-19 infection.

## 1. Introduction

Thrombocytes or platelets are cells produced by megakaryocytes. They are small blood cells that are responsible for physiological responses like hemostasis, thrombosis, immune responses, and wound healing [1]. The megakaryocyte is the type of myeloid cell that is mainly found in the bone marrow, and a few of them are found in the lung. Megakaryocytes are developed from pluripotent hematopoietic stem cells (HSC). These HSCs are differentiated into burst-forming cells and colony-forming cells along with the expression of CD34 antigen, and these cells act as precursors for megakaryocytes [2]. The thrombocyte precursors are produced from the megakaryocytes through thrombopoiesis [3]. Thrombopoietin (TPO) is a glycoprotein that enhances the megakaryocytes to differentiate into thrombocytes through the binding with c-Mpl, which is present in the megakaryocytes. This protein is translated by TPO mRNA which is mainly expressed in the hepatic cells [4].

At the last stage of differentiation, megakaryocytes move to the perivascular microenvironment where they attach with endothelial cells called proplatelets, which are differentiated into thrombocytes [3]. The presence of two types of megakaryocytes was observed in the bone marrow as well as in the lungs [5].

Normally, when the individual is affected by any disease or disorder, the individual must be undergone a full blood count, because the full blood count is an indicative assessment of the normal function of the body. Gentle thrombocytopenia, regularly joined with lymphocytopenia, is an average of the most intense viral diseases yet is neither adequately touchy nor explicit to dependably recognize viral from bacterial or parasitic microbes [6]. The platelets express surface receptors such as Toll-like receptors, lectins, and integrins that allow them to interact with the pathogens directly and form the reason behind the fact that platelets can suppress viral infection [7]. Platelet factor 4 is a platelet kinocidin that is released abundantly during the time of infection, while platelet-viral interaction occurs, and this platelet factor 4 is responsible for inhibiting the broad spectrum of HIV-1 [8]. Except for viral hemorrhagic fevers and uncommon instances of serious spread viral diseases, infection-incited thrombocytopenia does not prompt critical dying, seldom requires thrombocyte bonding, and is consequently effectively excused as clinically insignificant. Be that as it may when the connection between thrombocytes and viral contamination is concentrated all the more intently and in bigger review populaces, significant discoveries arise which shed light on beforehand unnoticed parts of viral infections [9].

Arising irresistible infections consistently represent a danger to people alongside plant and creature life. The severe acute respiratory syndrome-coronavirus 2 (SARS-CoV-2) is also one such viral contamination that started in Wuhan city of the Republic of China in December 2019. Presently, it has turned into a pandemic. SARS-CoV-2 has contaminated more than 500 million individuals around the world and taken 6,261,708 living souls (WHO, 2022). It was named first “WH 1 Human CoV” and later changed to the 2019 Novel CoV. Researchers have set up it as a zoonotic viral illness that arose out of Chinese horseshoe bats, which do not foster serious contamination [10].

This review mainly focuses on the findings that the lung is also one of the sites for thrombocytopoiesis and will discuss how thrombocytes biogenesis in the lung will be maintained under normal conditions and how the process will be changed in SARS-CoV-2 patients especially focusing on thrombocytopenia.

## 2. Methodology

The data collected for this review are taken from PubMed search, PubMed Central, ScienceDirect, and some journals like Nature and Cell through Google surfing. About 40 papers are from PubMed Central, 35 papers are from PubMed search, and 20 papers are searched from ScienceDirect and Google surfing. The search for a paper is mainly based on the keywords of the title. “Thrombocytes,” “c-Mpl and TPO interaction,” “thrombocyte biogenesis in lungs,” “SARS-CoV-2,” and “thrombocytopenia” are the keywords that are used for searching the review.

## 3. Structure and Genetics of Thrombopoietin

TPO belongs to the member of the cytokine family which is essential for the regulation of thrombocyte formation from megakaryocytes by binding with its c-MPL. TPO functions an essential role in the formation of thrombocytes [11], which was proved by the findings of Gurney et al. [12] the result of this study shows that TPO^−/−^ and MPL^−/−^ mice have approximately 85% a low number of thrombocytes and megakaryocyte when compared with normal lineage. TPO is one of the hematopoietic growth factors, which was first isolated by Kelemen in 1958 who proposed that TPO is involved in thrombocyte production [13]. The liver is the predominant site for the secretion of TPO. The liver secretes TPO as a precursor protein containing 353 amino acids with a molecule weight of 36 KDa [14]. After the posttranslational modification, 21 amino acids are spliced, and the rest of the 332 amino acids enter into the glycosylation process and at last produce a glycoprotein with a molecular weight of 95 KDa on SDS-PAGE [15].

Human TPO (hTPO), a glycoprotein contains two different regions—the N-terminal region and the C-terminal region [16]. The N-terminal region contains 153- residues and shares about 23% similarity with human erythropoietin (EPO), and both of them are homologous with each other. The N-terminal region is essential for the binding of receptors and carrying out the signal transduction process. The C-terminal region contains 179- residues among this proline, glycine amino acids are predominant, and also, this region contains six N-linked glycosylation sites [17]. Even though some of the findings indicate its role in secretion and protection from the lyses of protein, its function is not identified. The C-terminal region does not involve in the process of receptor binding and this region is not as much conserved terminal as the N-terminal region [18]. A study by Feese et al. [16] finds out that the receptor-binding domain of human TPO (*hTPO*) is about 2.5A^0^ in resolution by crystallization of neutralizing Fab fragment.

The gene which encodes the thrombopoietin protein is the *TPO* gene. It has six coding regions and five noncoding regions and the length is about 6.2 Kb [19]. The chromosomal locus of the *hTPO* gene is 3q26-q28 [20]. The transcription of mRNA of the *hTPO* gene predominantly takes place in the liver and kidney, and a small amount of mRNA is seen in the lungs, spleen, and bone marrow [21]. *EVI1* canonical sequence and ETS family transcription factors are located in the flanking region of the 5′ [22], and these are important for the promotion of activation of *TPO* gene expression [23].

## 4. Biology of c-Mpl Receptor

*c-Mpl* is the gene that encodes for the receptor of TPO. In mice, *c-Mpl* expression was found in the spleen and fetal liver after the examination of organs such as the brain, liver, salivary gland, spleen, kidney, testis, thymus, and fetal liver, by using the technique called northern blot analysis [24].

The c-Mpl of humans has 635 amino acid transmembrane proteins. It is one of the members of the type I cytokine receptor subfamily. c-Mpl has two cytokine receptor modules (CMR) whereas other subfamily members have one CMR [25]. The length of each CMR is about 200 amino acids. The N-terminal has four conserved cysteines and the C-terminal region has a WSXWS motif. Studies by Sabath et al. [26] show that TPO, combined with CRM which is in the distal region, is involved in the suppression of *c-Mpl* expression. Human c-Mpl is predominantly present on the surface of hematopoietic stem cells (HSC) and thrombocytes [27]. c-Mpl (both mRNA and protein) is seen on CD34^+^ CD38^−^ cells, thrombocytes, and human megakaryocytes [28]. Approximately 12,000 c-Mpl receptors are present on the surface of megakaryocytes whereas thrombocytes contain about 25-200 [29]. The gene encodes for *c-Mpl* is mapped on human chromosome 1 at the position of 1p34. This gene has 12 exons, and the length of the gene is about 17 Kb. Similarly, *the c-Mpl* of the mouse is located on chromosome 4 [30]. The promoter region of *the c-Mpl* gene has a binding site for ETS (erythroblast transformation specific), SP1 (specificity protein 1), and GATA-1 (GATA binding protein 1), which induce the transcription of *the c-Mpl* gene [3]. The arrangement of the genes and the splicing sites are as much similar to the *c-Mpl* of humans. In the human *c-Mpl* gene, alternate splicing at the 3′ end leads to the production of three different types of mRNA. The first one is the P-form which encodes the full-length protein along with the transmembrane domain, and it has almost 122 cytoplasmic residues. The next mRNA species of human *c-Mpl* is K-form, which is formed by splicing beyond the exon 10 splice site. This K-form has 66 cytoplasmic residues, and this form does not involve biological activity. The last one is the *Mpl-tr* form, which is commonly found in murine as well as in humans. And this mRNA is formed by splicing exon 8 directly to exon 11, which leads to the elimination of the Juxta membrane motif WSXWS and the transmembrane domain [31]. Among these three forms, P-form is the predominant one [32].

## 5. Interaction of Thrombopoietin and c-MPL

Interaction between TPO and c-Mpl induces the signal for regulating thrombocytes count in our body. Thrombopoiesis is a complicated process, where HSCs are differentiated into promegakaryocytes. These promegakaryocytes differentiate into megakaryocytes, the precursors of thrombocytes [33]. Megakaryocyte precursors proliferate and mature into megakaryocytes. Megakaryocytes are giant cells with multilobulated polyploid nuclei, and they extend their long thin cytoplasmic processes known as proplatelets into the bone marrow sinusoids where they release thousands of thrombocytes. These thrombocytes have entered circulation [34].

Platelet production undergoes two simultaneous processes such as megakaryocytopoiesis and thrombopoiesis. Here, megakaryocytopoiesis, a process where the megakaryocyte is produced, is controlled by the TPO. The TPO is largely produced by the liver [35]. TPO binds as a ligand with the member of the hematopoietic cytokine receptor super family called c-Mpl and induces megakaryocytopoiesis and thrombopoiesis [36]. The level of TPO production is inversely proportional to thrombopoiesis. Because the TPO is cleared during thrombocyte production when the thrombocyte production rate is high, more TPO is catabolised as a result, a low level of TPO is present in the circulation. In contrast, during the low rate of thrombocyte production, the TPO clearance is low, and a decreased level of catabolism of TPO takes place, so the level of TPO raises [37].

**Figure 1:**
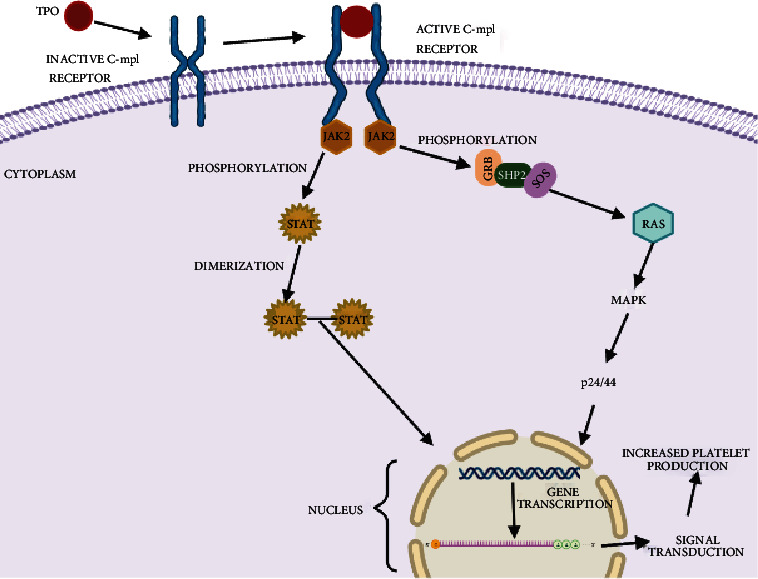
Interaction of TPO and c-Mpl receptor.

When TPO binds with its receptor c-Mpl, homodimerization of the receptor occurs, which leads to the activation of JAK2 [38]. Activated JAK2 then phosphorylates tyrosine residues which are present in the receptor; as a result, a series of intracellular signals arise from various signalling molecules such as SHC, GRB2, SOS, VAV, and CBL leading to the thrombopoiesis [39]. Among these important intracellular signalling pathways are JAK/STAT, MAPK/ERK, and PIK/AKT [40, 41]. From the above signalling pathway, JAK/STAT and MAPK function an important role in the proliferation and maturation of megakaryocyte progenitor whereas the PI3K/AKT pathway is essential for the progression of the cell cycle [42] (Figure 1).

Homodimerization of the c-Mpl occurs when TPO binds to it. As a result, JAK2 kinases activate. The activated JAK2 kinases and Mpl receptors utilise phosphorylation. Then, the tyrosine residues of receptor phosphorylate by the activated JAK2 lead to the involvement of many proteins including SHC homology or phosphotyrosine binding motifs. JAK2 then activates STAT, leads to the translocation from cytoplasm to the nucleus, and involves in the transcription of genes such as BCL2L1 and cyclin D1. These genes are responsible for extending cell life [43]. TPO binding also stimulates the PI3K and MAPK pathways which are all essential for cell survival especially megakaryocytes to activate the rate of thrombopoiesis [44].

## 6. Involvement of Lungs in Thrombopoiesis

Even though there is much evidence that shows that the lung is also a site for thrombopoiesis, till now, this concept is not accepted because of its indirect and unknown mechanism. The findings of Zucker-Franklin and Philipp [45] involves the reexamination of the hypothesis of Levine by conducting process such as phlebotomy and instrument of thrombopoietin in mice and analysing the lung specimen using ultrastructural analysis to identify the thrombopoiesis in the lungs. The result of this experiment showed the presence of intact megakaryocytes and megakaryocyte fragments in the pulmonary region of the animal.

The work of Lefrançais et al. [46] proved the hypothesis given by Howel and Dusche that the lung acts as a reservoir for thrombopoiesis. Here, Lefrançais et al. [46] show the microcirculation of megakaryocytes in the lungs of mice and also discovered that almost 50% of total thrombocyte production takes place in the lungs. To carry out this experiment, they used a PF4-mTmG reporter mouse as a model, where PF4-Cre stimulates the expression of GFP (green fluorescence protein) on the membrane of the megakaryocyte and thrombocyte. Therefore, they proved that the lung is also involved in the biogenesis of thrombocytes in mammals [47]. So, the above experiment done by Lefrançais et al. [46] showed that the lungs have two types of megakaryocytes, based on their origin. One is extra pulmonary origin includes megakaryocytes from bone marrow and spleen, and another one is residing inside the lungs itself, but their origin and function are unknown [46].

Further findings of Yeung et al. [48] strongly prove the involvement of the lungs in the process of thrombopoiesis. Here, they discovered that lung megakaryocytes are essential for thrombocyte production when the regulation of thrombopoiesis by the bone marrow is affected. They proved that lungs have megakaryocytes by the application of the single-cell RNA sequencing technique and the findings of Yeung et al. [48] also proves that the lung megakaryocytes are also involved in immunity and inflammation. Furthermore, Pariser et al. [49] show that megakaryocytes present a large amount in the extra vascular region of the lungs and about 30% of megakaryocytes are seen in the intravascular region of the lungs. They also noted the expression of CD11C after using the technique of single-cell RNA sequencing.

**Figure 2:**
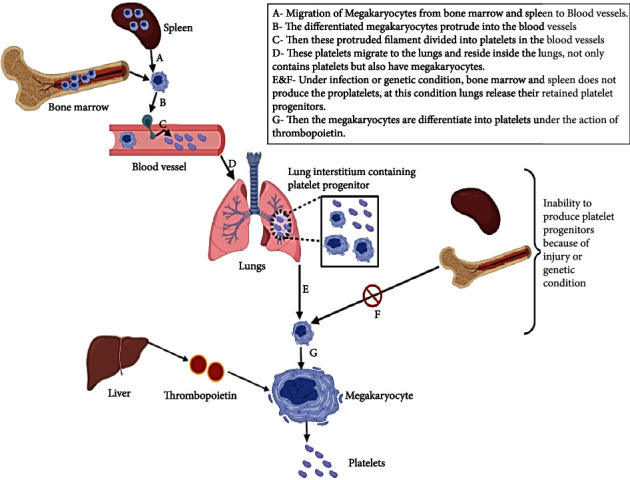
Biogenesis of thrombocytes in lungs.

Megakaryocytes have also been seen in the lungs of humans, and they increase their number during lung disorders and air-borne diseases [50]. The study by Kaufman [51] shows that the right atrial region of 23 patients who are all carrying out diagnostic cardiac catheterization contains megakaryocytes. At last, Kaufman [51] highlighted that the megakaryocytes that reside inside the lungs do not participate in the thrombopoiesis unless the deficiency of megakaryocytes which was produced by the bone marrow occurs. The regulation of thrombocytes takes place in two phases, one is one bone marrow, and another one is taking place in the lungs (Figure 2). But the regulation mechanism that takes place in the lung is still unclear [52].

## 7. Viral Infection and Thrombocytopenia, Interconnected?

The level of thrombocytes decreases when the lungs are damaged because of some viral infection that targets the lung cells for their replication [53]. As we know, the lung is also one of the sites for thrombopoiesis, so lung infection also interconnects with thrombocytopenia [54]. During infection which is caused by influenza, thrombocytopenia is also associated. [55]) identified that patients who have been infected with influenza have low thrombocyte counts compared to nonaffected people. The experiment is conducted on the ferret where that have been infected with the influenza A strain. As a result, they develop thrombocytopenia. And also, this influenza virus binds to alpha glucans which are present on the cell surface of thrombocytes through the binding of sialic acids, thereby affecting the platelets. Jansen et al. [56] explained the mechanism of clearance of thrombocytes by the interaction of sialic acid between thrombocytes and the influenza virus. To initiate the removal of thrombocytes, the influenza viruses are phagocytosed by thrombocytes. As a result, the sialic acids are removed by the enzyme called neuraminidase, produced by the virus. This clearance of thrombocytes from the circulation leads to thrombocytopenia in the infected ferret model. They also identified the interrelationship between thrombocytes counts and the pathogenicity of the virus which is ranging from 0% in influenza A/H3N2 virus to 22% in the pandemic influenza A/H1N1 virus and lasts up to 62% in the more infectious strain A/H5N1.

Another important viral infection that is involved in thrombocytopenia is the measles virus. They are highly virulent viruses that affect humans. This virus first targets the respiratory tract and at last attacks the lymphoid organs, where they infect the lymphocytes [57]. As it shows thrombocytopenia in the postinfection stage, the lung damage which is caused by the virus plays an important role [58]. Next to the measles virus, parvovirus B19 also interconnects with thrombocytopenia. Parvovirus B19 is a viral infection they are characterized by the presence of mild rash illness. These are commonly found in pregnant women and children [9]. In pregnant women, these viruses cause a condition called hydrops fetalis in the developing fetus [59]. Fetal parvovirus B19 induces thrombocytopenia and anaemia in the fetus, because of its external stimulus on erythroid progenitor cells and megakaryocytes [60, 61].

In addition to the above-mentioned viral infections, the SARS-CoV-2 places a major area in the interconnection among the lung, thrombocytopenia, and their infection of the lungs. It creates a pandemic across the world. It is a multiorgan attacking disease [62]. In the severe COVID-19 condition, the plasma level of TPO is increased, but inversely, the expression of gene *c-Mpl*, the receptor of TPO, is downregulated. As a result, thrombocytopenic condition occurs in those patients [63]. The mechanisms involved in the pathogenesis of viral infection that leads to thrombocytopenia thereby affecting the lungs are given in Table 1.

## 8. SARS-CoV-2 the Overview

The term corona was taken from a Latin word that means crown. When they are observed under an electron microscope, the crown-like spikes are present on the surface, so they are named coronavirus [64]. These belong to the subfamily of Coronavirinae which comes under the Coronaviridae family. Four genera fall under the Coronavirinae subfamily; they are alpha coronavirus, beta coronavirus, delta coronavirus, and gamma coronavirus. From the above-mentioned genera, the SARS-CoV-2 strain belongs to beta coronavirus genes [65]. They have enveloped virus that consists of a single-stranded, positive-sense RNA genome which has approximately 32 kilobases in length. The genome of coronavirus has a 5′ cap and a 3′ polyadenylate tail, because these virus can produce the enzyme for their replication in the host [66]. They are transmitted based on zoonotic mode, i.e., transmission occurs from animals to humans. Normally, these viruses have been mainly found in mammals and are rarely seen in birds [67]. Currently, this virus also spread to humans, thereby causing to initiate an epidemic, and they are transmitted through zoonotic transmission. In the COVID-19 case, bats are the primary carrier of viral transmission. Here animal-human acts as a barrier, so it creates a pandemic across the world. But, human coronaviruses were identified first in the period of 1960s [68]. Hence, the coronavirus is not the new one for us.

COVID-19 is transmitted via animal-human or human-to-human or nosocomial-related routes. Because of their virulent transmission, it creates a pandemic status. CoV-2 is highly contagious and causes respiratory disorders. The SARS-CoV-2 shares nearly 80% of a similar genome with SARS-CoV and also SARS-CoV-2 binds with angiotensin-converting enzyme-2 (ACE-2) receptor as similar to SARS-CoV for the cell entry and further replication [69]. Spike protein of CoV-2 is trimeric which is about 180 KDa in length and has S1and S2 subunits, where S1 is essential for its attachment and S2 is for its fusion [70]. The data provided by Ou et al. [70] shows that the stability of SARS-CoV is higher than that of SARS-CoV-2. The study by Wrapp et al. [71] explained that the SARS-CoV-2 attaches with the ACE-2 receptor through the peptidase domain with high specificity. So, from the above information, the receptor-binding domain which is present in the S1 subunit of SARS-CoV-2 plays an important role in binding with ACE-2 through the peptidase domain; thereby, the viral genome enters into the cell for their replication [72, 73].

## 9. Structure of SARS-CoV-2

Spike protein (S) of coronavirus is large and class I transmembrane protein of the virus. The size varies from the range of 1160 to 1400 amino acids [74]. These proteins are seen on the cell surface of the virus, and it acts as a connecting bridge between the host cells and the viral genome for their replication. Importantly, these proteins induce the host's immunity, and the domain of the S protein is divided into S1 and S2 [75]. The former one (S1) is responsible for the binding of receptors, and the latter one (S2) is for complete fusion [76]. A comparison of the sequence of S protein between SARS-CoV and SARS-CoV-2 shows 17 replacement mutations. Because of this mutation, the nature of the binding domain varies between them [77].

Another protein is the M protein. It is a viral protein that is essential for the solid and definite shape of protein [78]. M protein is made up of three transmembrane domains are bound outside the virion protein is made up of three transmembrane domains that are bound outside the virion by an amino terminus and inside the virion by a carboxy terminus [79]. The important finding is that there is no substitution mutation between SARS-CoV-2 and SARS-CoV [80]. The next important protein is the E protein which is paradoxical and small in length among the other proteins [81]. It plays an important role in the pathophysiology and replication of viruses. If the E protein is not present, it will change the severity and pathogenicity of coronavirus [82]. The analysis of the sequence of E protein shows a null difference between the SARS-CoV and SARS-CoV-2 [80]. The last one is the N protein which is essential for the production of the viral genome and also increases the transcription process [83]. Here, the analysis of the sequence of N protein between the SARS-CoV-2 and SARS-CoV reveals that there is five amino acid substitution that takes place between them [80].

## 10. COVID-19 and Thrombocytopenia

Zhang and their colleagues showed that the SARS-CoV-2 patients have a high mean thrombocyte volume that paralleled with a low thrombocyte overall count. They also found out that the thrombocytes have ACE-2 which is a receptor that plays an important role in the COVID-19 virus for their invasion into the cells, because the S1 domain of spike protein enters the cells through ACE-2 for their replication [84]. The study conducted by Hoffmann et al. [85] explained the transmembrane protease serine 2 (TMPRSS2), which primer the cell entry of SARS-CoV-2 for their replication. Zhang et al. [84] find out that the thrombocytes have higher expression of TMPRSS2 than the human caco-2 cells, human calu-3 cells, and mouse lung tissue after the analysis of RNA level and protein level. This confirms that thrombocytes express the ACE-2 and TMPRSS2 which is essential for the cell invasion of SARS-CoV-2 [84]. So, the study of Zhang et al. [84] finds out that thrombocytes activation is increased in COVID-19 patient that is proved by high expression of *α*IIb*β*3 and P selectin activation, and also, they find out that the MAPK (mitogen-activated protein kinase) pathway along with ACE-2 is essential for the promotion of thrombocytes formation which is associated with COVID-19 infection [84].

Comparison between the COVID-19 patient and normal patients by Zaid et al. [86] also shows that thrombocytes have the mRNA of ACE-2 [86]. The analysis done by Koupenova et al. [87] shows that the SARS-CoV-2 is internalised within the thrombocytes either attached to the microparticle or directly. As a result, programmed cell death of thrombocytes occurs, and that leads to the condition called thrombocytopenia. Here, necroptosis happens for the cell death of platelets which is a caspase-independent that is based on the initiation of MLKL (mixed lineage kinase domain-like protein) phosphorylation [88]. Barrett et al. [89] identified the marker for the activity of thrombocytes which are correlated with the severity of illness and cause of death. D-dimer and C-reactive protein (CRP) are the biomarkers associated with SARS-CoV-2 and the activity of the thrombocytes. There is no direct evidence of an invasion of the virus into the thrombocytes, but the above findings have explained the mechanism of SARS-CoV-2 invasion into the thrombocytes. However, the mechanism is still unclear.

## 11. Other Possible Mechanisms

Thrombocytopenia occurs in COVID-19 patients because SARS-CoV-2 targets the bone marrow where the proplatelets are synthesized [90]. The antigens of SARS-CoV-2 and HCoV-229E are identical. Hence, the pathogenicity of SARS-CoV-2 and HCoV-229E is similar where HCoV-229E targets the CD13 that acts as the receptor for HCoV-229E, presents on the cell membrane of the intestine, kidneys, or lung. This CD13 acts as a marker for white blood cells (WBC) and is enormously present in the lymphocytes and thrombocytes. As SARS-CoV-2 follows the mechanism of HCoV-229E for transmission, it enters the thrombocytes through CD13 receptors and thus stimulates the apoptosis of thrombocytes, leading to thrombocytopenia [91]. Zaid et al. [86] performed a blood analysis on 33 severely and critically affected COVID-19 patients, and they found the synthesis of granulocyte-macrophage colony-stimulating factor (GM-CSF) and interleukin-6 (IL-6) because of the overactivated T-cells after the infection of COVID-19. This in turn induces the CD14^+^ CD16^+^. The activated CD14^+^ CD16^+^ induces the increased production of IL-6 destroying the thrombocyte progenitor that is present in the bone marrow. As a result, the thrombocyte count in the blood decreases leading to the thrombocytopenic condition [92].

The infection of SARS-CoV-2 may lead to an increased level of autoantibodies which results in the breakdown of thrombocytes by the activity of the immune system. Still, the pathogenesis mechanism of COVID-19 is unknown, but it may follow the infection mechanism of human immunodeficiency virus-1 (HIV-1) as both of them are retrovirus. In HIV-1, it induces the immune complex which contains antithrombocyte membrane GPIIIa49-66 Ig G antibodies. Then, HIV-1 GP160/120 antigen interacts with GPIIIa49-66 Ig G and settles on the thrombocyte surface. Hence, the thrombocytes are targeted by the immune cells and as a result, they are destroyed. This leads to a massive decrease in thrombocyte count that leads to thrombocytopenia [93]. SARS-CoV-2 may also follow the same mechanism, but till now, a clear mechanism is not identified [91].

## 12. Conclusion

The lung releases its thrombocyte from the interstitium when the level of the thrombocyte falls below the normal. During the COVID-19 condition, the level of thrombocyte falls below the normal level of thrombocytes because of ACE-2 action. Therefore, upcoming studies that concentrate on the association of the ACE-2 mechanism with the decrease in thrombocyte counts are to be included. Therefore, from this review, it is informed that COVID-19 patients may develop thrombocytopenia as a post-COVID symptom. From this, we can understand that the lung is also an important vital organ that functions an important role in the hemostasis of human physiology. It is concluded that there is a variation in thrombocyte count between normal people and COVID-19-affected people. Hence, COVID-19 influences the development of thrombocytopenia in patients.

## Figures and Tables

**Table 1:** The table representing the pathogenesis of viral infections involving lung and platelets.

Viral strains	Pathogenesis involving lung and thrombocytopenia	References
Influenza virus (H1N1)	(i) Binds to alpha glucans on the platelet surface and affects the platelet homeostasis (ii) The interaction of sialic acid between the platelets and the lungs, mediates the clearance of platelets	[56]
Measles virus	(i) The virus upon infection causes thrombocytopenia as it causes damage to the lungs during the time of infection	[57]; [58]
Fetal parvovirus B19	(i) The thrombocytopenia is induced during the time of infection due to the external stimulus of the virus on erythroid progenitor cells and megakaryocytes	[60]; [61]
SARS-CoV-2	(i) The plasma level of thrombopoietin (TPO) increases which in turn decreases the expression of the TPO-receptor by means of the *C-mpl* gene that ends up causing thrombocytopenia	[63]

## Data Availability

The authors confirm that the data supporting the review are available upon reasonable request with the corresponding author.
